# The role of traditional obesity parameters in predicting the number of stenosed coronary arteries (≥ 60%) among patients undergoing cardiac catheterization

**DOI:** 10.1038/s41598-022-17517-0

**Published:** 2022-08-15

**Authors:** Audai A. Hayajneh, Islam M. Alhusban, Mohammad Rababa

**Affiliations:** Adult Health-Nursing Department, Faculty of Nursing, Jordan University of Science and Technology, P.O. Box: 3030, Irbid, 22110 Jordan

**Keywords:** Acute coronary syndromes, Obesity

## Abstract

The correlation between obesity and coronary artery disease (CAD) has been well-documented in the literature. Body mass index, waist–height ratio, waist–hip ratio, body adiposity index, body shape index, waist circumference, and hip circumference are traditional obesity parameters used to measure obesity. This study aimed to investigate the role of these traditional obesity parameters in the prediction of the number of stenosed coronary arteries (≥ 60%) among patients undergoing cardiac catheterization. A descriptive cross-sectional study was conducted among 220 hospitalized patients undergoing cardiac catheterization in two hospitals in Jordan. Bivariate Pearson’s correlation and forward linear regression analysis were used in the current study. Hip circumference was identified as being the best predictor of CAD (r = 0.5), with the best cut-off value of 103 cm (sensitivity = 0.92, specificity = 0.58). Hip circumference had significant regression levels with the number of stented coronary arteries (P = 0.002) and the number of severe stenosed coronary arteries (P = 0.04). The second-best obesity parameter in predicting CAD was waist circumference (r = 0.4), with a cut-off value of 0.95 m (sensitivity = 0.76, specificity = 0.68). High-sensitivity C-reactive protein (HS-CRP), triglycerides, and smoking had significant positive correlations with the number of stented coronary arteries (P < 0.05). Hip circumference of ≥ 103 cm, increased serum level of triglycerides, HS-CRP, and being a smoker are all factors which can predict CAD or the risk of developing it.

## Introduction

Obesity is conceptually described as the excessive accumulation of fatty tissue across the human body, predisposing patients to several health risks^1^. These health risks include the increase in total cholesterol, low density lipoprotein (LDL), triglycerides, and insulin resistance, which predispose people to various heart, lung, kidney, and brain diseases^2^. The WHO^1^ operationally classifies obesity by body mass index (BMI), which is the result of dividing weight in kilograms by the square of height in meters, into obese stage I (BMI of 30–34.9), obese stage II (BMI of 35–39.9) and obese stage III (BMI of 40 or more).

Obesity is a predisposing risk factor for dyslipidemia, coronary artery disease (CAD), chronic obstructive pulmonary disease, and diabetes mellitus^3^. Obesity increases LDL and triglycerides and decreases high density lipoprotein (HDL), which leads to the accumulation of fat on the interior wall of the arteries (intima) and causes the formation of fat plaque (atherosclerosis)^4^.

Coronary artery disease is conceptually defined as the narrowing or occlusion of one or more coronary arteries, resulting from the deposition of cholesterol under the epithelium layer that encloses the tunica intima (the inner most layer of the artery). This build-up of cholesterol forms fat plaque (atherosclerosis), where the inflammatory process takes place. Epithelium injury to the coronary artery’s inner wall layer occurs when the narrowing percentage increases (due to atherosclerosis), which leads to the increase in pressure against the coronary artery^5^.

There are other risk factors for CAD, including anxiety, depression, sedentary lifestyle, and smoking. Anxiety is conceptually defined as the anticipation of a future danger or negative event, accompanied by feelings of dysphoria or physical symptoms of tension^6^. Anxiety is also operationally defined according to a score of 11–21 using the Hospital Anxiety and Depression Scale (HADS)^7^. The positive association between anxiety and CAD was evidenced in the study of Celano et al.^8^.

There are numerous traditional parameters used to measure obesity^9–14^. Body mass index (BMI), waist-height ratio (WHtR), waist-hip ratio (WHR), body adiposity index (BAI), body shape index (BSI), waist circumference (WC), and hip circumference (HC) have been used to measure obesity and have been correlated with the risk factors for CAD (i.e., hyperlipidemia, hyperglycemia, hypertension)^15^.

Previous prospective descriptive studies^9–14^ have investigated the relationships between traditional obesity parameters (WHtR, WHR, BAI, BMI, BSI, WC, and HC) and CAD or its risk factors (i.e., hyperlipidemia, hyperglycemia, smoking, and age). However, these studies have reported contradictory findings with regards to the best obesity parameter for predicting CAD and its risk factors. Further, these studies did not compare between all of these obesity parameters together in terms of their role in predicting CAD or the number of stenosed coronary arteries (≥ 60%) and number of stented coronary arteries. Therefore, the present study aimed to compare between the seven traditional obesity parameters (WHtR, WHR, BAI, BMI, BSI, WC, and HC) in terms of their role in predicting the number of stenosed coronary arteries (≥ 60%) among patients with CAD undergoing cardiac catheterization.

## Methods

### Research design

This study adopted a correlative descriptive cross-sectional design with self-report questionnaires used to collect data from patients before undergoing cardiac catheterization. The questionnaires were completed by well-trained research assistants. The use of a cross-sectional design allowed for collecting data in a feasible manner and reaching a large and varied sample of patients undergoing cardiac catheterization in Jordan.

### Setting and sample

The study setting included a university-affiliated hospital and a private hospital, both of which were located in Jordan and were major referral centers in the selected geographical region. Convenience sampling was used to recruit 172 patients undergoing cardiac catheterization in the selected hospitals. G*Power was used to calculate the required sample size, given F test as family test and linear hierarchical multiple regression with an effect size of 0.15, alpha error probability of 0.05, power of 0.8, and number of predictors of 25. To account for any possible dropouts, an additional 25% of the required sample size was added. In total, 220 participants were recruited.

The inclusion criteria included being a Jordanian male or female aged 18 years or over and being a hospitalized patient undergoing either elective or urgent cardiac catheterization. Patients were excluded if they had severe organ diseases, such as severe liver disease (bilirubin > 5 mg/dl, international normalized ratio (INR) ≥ 1.5)^16^ and renal failure (increase in serum creatinine above 2.8 mg/dl)^17^, were pregnant, had undergone coronary artery bypass graft surgery, had autoimmune diseases, or were immunosuppressed patients (e.g. patients who had undergone organ transplant or who had cancer).

### Data collection

The independent variables were the obesity parameter measures. Weight, height, WC, and HC measures were recorded by well-trained registered nurses who had completed three 1-h training sessions held by the primary researcher. Data collection took place during the admission assessments held before cardiac catheterization, with the data collection period lasting from March 18, 2021 to July 18, 2021.

### Instruments

Information about the socio-demographic and health variables of the participating patients was collected from the patients themselves or from their medical files. These variables included age, gender, education, marital status, income, employment, smoking status, blood pressure, daily activity (measured by a pedometer device for counting steps), LDL, HDL, triglyceride, random blood sugar (RBS), and hemoglobin A1c (HbA1c).

Height measurements were taken using a standard stadiometer with shoes taken off, and the measurements were rounded to the nearest 0.1 cm. Weight was measured using a rigid measurement device and rounded to the nearest 0.1 kg, with the participants wearing their hospital gowns. Waist circumference was measured to the nearest 0.1 cm at the midpoint between the lowest rib margin and the level of the anterior superior iliac crest using a flexible anthropometric tape. Hip circumference was measured to the nearest 0.1 cm at the greatest protrusion of the gluteal muscles, whilst WHR and WHtR were calculated as WC/HC and WC/height, respectively. Body shape index was calculated using the formula (WC (cm))/([BMI] ^2/3^) × ([Height (m)]^0.5^), BMI using the formula (weight (kg))/[height (m) ]^2^, and BAI using the formula (HC (cm))/[height (m)]^1.5^ − 18.

Anxiety and depression levels were analyzed as continuous variables (score from 1 to 21) and measured using the Hospitalized Anxiety and Depression Scale (HADS). The HADS is a psychometric tool developed by Zigmond et al.^7^ to measure anxiety and depression in hospital. The internal consistency of the scale has been reported as good, with Cronbach’s alphas of 0.87 for anxiety and 0.81 for depression^18^. Terkawi et al.^19^ translated the original HADS into Arabic and reported Cronbach’s alphas of 0.83 for anxiety and 0.77 for depression. In our study, the Arabic version of the HADS^19^ was used to measure anxiety and depression in patients due to undergo cardiac catheterization. The total scale scores were classified as follows: 0–7 = normal, 8–10 = borderline abnormal (borderline case), 11–21 = abnormal (i.e., has anxiety or depression).

The numbers of severe stenosed coronary arteries (≥ 60%) were determined using the system metric tool by the cardiologists who performed the cardiac catheterization procedures. The numbers of stented coronary arteries (including only the current implemented stents) were also determined by the cardiologists who performed the cardiac catheterization procedures.

### Data analysis

Means and standard deviations were used to describe the continuous variables, such as age, whilst frequencies and percentages were used to describe the categorical variables, such as gender. Bivariate Pearson’s correlation was employed to assess the correlation (the strength of the relationship between variables) between “obesity parameters, socio-demographic variables, and health variables” and CAD (i.e., number of severe stenosed coronary arteries (≥ 60%) and number of stented coronary arteries). To test the relationship between the categorical variables and the dependent variables, “t test” was used for variables with two categories (e.g., gender) and “ANOVA test” was used for variables with more than two categories (e.g., employment status, marital status, and educational level).

Forward linear regression was employed to assess the ability of the obesity parameters, socio-demographic variables, and health variables to predict CAD (i.e., the number of severe stenosed coronary arteries (≥ 60%) and number of stented coronary arteries). A significance level (P value) of 0.05 was set for the statistical analyses. The Statistical Package for the Social Sciences (SPSS) software version 25 was used to analyze the study data.

### Ethical considerations

After obtaining approval to conduct the study from the Institutional Review Board at Jordan University of Science and Technology (Ref#20210093) and the selected hospitals, signed informed consent were obtained from all participants prior to their participation. These forms outlined the participants’ rights, including their right to withdraw from the study. The research assistants then collected data from participants who met the eligibility criteria. All methods were performed in accordance with the relevant guidelines and regulations.

## Results

Around two-thirds (73.2%) of the patients were male, and the majority of the patients (62.3%) were married. The average age of the participants was 49.9 years (SD ± 11.7), with ages ranging from 24 to 90 years. Based on serum level of LDL and high sensitivity C-reactive protein (HS-CRP), the majority of the patients were classified as being at high risk for CAD (55.9% and 65.9%, respectively). Based on serum level of HDL and triglycerides, the majority of the participants were classified as being at borderline level for CAD (64.1% and 39.5%, respectively). Furthermore, almost half of the participants (46%) were diabetic (HbA1c ≥ 6.5), around two-thirds (72.7%) had anxiety, and around half (45.5%) had depression (see Table 1).

**Table 1:** Socio-demographic and health variables of the study participants (*N* = 220).

	N (%)	Mean (SD)	Median (Min, Max)
**Gender**			
Male	161 (73.2%)		
Female	59 (26.8%)		
**Age (years)**		49.9 (11.4)	49 (24, 90)
**Marital status**			
Single	53 (24.1%)		
Married	137 (62.3%)		
Divorced	15 (6.8%)		
Widowed	15 (6.8%)		
**Employment**			
Employed	108 (49.1%)		
Unemployed	68 (30.9)		
Retired	44 (20%)		
**Educational level**			
Illiterate	10 (4.5%)		
Primary school education	40 (18.2%)		
High school education	72 (32.7%)		
Bachelor’s degree	56 (25.5%)		
Master’s degree	27 (12.3%)		
Doctoral degree	15 (6.8%)		
**Smoking (cigarette/day)**		26 (14)	25 (0, 60)
Nonsmoker (0)	84 (38.2%)		
Light smoker (< 10)	10 (4.5%)		
Moderate smoker (10–20)	72 (32.7%)		
Heavy smoker (> 20)	54 (24.6%)		
**Blood pressure**			
Normal (100–129/60–89)	44 (20%)		
Hypertension I (130–159/90–99)	80 (36.4%)		
Hypertension II (≥ 160/ ≥ 100)	96 (43.6%)		
**Daily activity (steps/day)**		3016 (1107)	3030 (789, 6100)
Sedentary life-style (< 5000 steps/day)	201 (91.4%)		
Borderline (5000–10,000 steps/day)	19 (8.6%)		
**Low density lipoprotein**		148 (18.8)	148 (102, 200)
Optimal < 100 mg/dl	22 (10%)		
Borderline (100–129 mg/dl)	75 (24.1%)		
High ≥ 130 mg/dl	123 (55.9%)		
**High density lipoprotein**		45 (4.8)	44 (34, 60)
Borderline	141 (64.1%)		
High	79 (35.9%)		
**Triglycerides**		188 (34.3)	180 (112, 277)
Optimal < 100 mg/dl	37 (16.8%)		
Normal 100–149 mg/dl	39 (17.7%)		
Borderline 150–199 mg/dl	87 (39.5%)		
High ≥ 200 mg/dl	57 (26%)		
**HS-CRP**		5 (1.4)	5 (2, 9)
Low risk	42 (19.1%)		
High risk	145 (65.9%)		
**HbA1c**		7.1 (0.86)	6.9 (5, 9)
Optimal	42 (19.1%)		
Borderline	76 (34.5%)		
Diabetic	102 (46.4%)		
**Anxiety (score of 1–21)**		11.8 (2)	12 (6, 16)
Normal	14 (6.4%)		
Borderline	46 (20.9%)		
Abnormal	160 (72.7%)		
**Depression (score of 1–21)**		10.7 (2.3)	11 (5, 15)
Normal	38 (17.3%)		
Borderline	82 (37.3%)		
Abnormal	100 (45.5%)		

*HS-CRP* high-sensitivity C-reactive protein, *HbA1c* hemoglobin A1c.

With regards to the numbers of stented coronary arteries and stenosed coronary arteries (see Table 2), around half of the participants (*N* 103, 46.8%) had one stented coronary artery, one third (*N* 60, 27.3%) had two stented coronary arteries, and a quarter (*N* 56, 25.5%) had no stent. Specifically, the left anterior descending coronary artery had the highest frequency of being stented (*N* 103, 46.8%), followed by the left circumflex coronary artery (*N* 53, 24.1%) and the obtuse marginal 2 coronary artery (*N* 1, 0.5%).

**Table 2:** Characteristics of CAD of the study participants (*N* = 220).

	Number	Mean (SD)	Median (Min, Max)
**Number of severe stenosed arteries**		1.15 (0.78)	1 (0, 3)
0 Stenosed artery	45 (20.5%)		
1 Stenosed artery	104 (47.3%)		
2 Stenosed artery	63 (28.6%)		
3 Stenosed artery	8 (3.5%)		
**Number of stented coronary arteries**		1.03 (0.74)	1 (0, 3)
0 stented artery	56 (25.5%)		
1 stented artery	103 (46.8%)		
2 stented artery	60 (27.3%)		
3 stented artery	1 (0.5%)		
**Left main coronary artery (LMCA)**			
Patent (no stenosis)	210 (95.4%)		
Stenosis (30–70%) without stented	1 (0.5%)		
Stenosis (60–100%) and stented	9 (4.1%)		
**Left anterior descending (LAD)**			
Patent (no stenosis)	84 (38.2%)		
Stenosis (30–70%) without stented	33 (15%)		
Stenosis (60–100%) and stented	103 (46.8%)		
**Left circumflex (LC)**			
Patent (no stenosis)	138 (60%)		
Stenosis (30–70%) without stented	35 (15.9%)		
Stenosis (60–100%) and stented	53 (24.1%)		
**Right coronary artery (RCA)**			
Patent (no stenosis)	172 (78.2%)		
Stenosis (30–70%) without stented	19 (8.6%)		
Stenosis (60–100%) and stented	29 (13.2%)		
**Diagonal 1 (D1)**			
Patent (no stenosis)	163 (74.1)		
Stenosis (30–70%) without stented	30 (13.6%)		
Stenosis (60–100%) and stented	27 (12.3)		
**Diagonal 2 (D2)**			
Patent (no stenosis)	217 (98.6)		
Stenosis (30–70%) without stented	2 (0.9%)		
Stenosis (60–100%) and stented	1 (0.5%)		
**Obtuse marginal 1 (OM1)**			
Patent (no stenosis)	207 (94.1%)		
Stenosis (30–70%) without stented	9 (4.1%)		
Stenosis (60–100%) and stented	4 (1.8%)		
**Obtuse marginal 2 (OM2)**			
Patent (no stenosis)	219 (99.5%)		
Stenosis (30–70%) without stented	0 (0.0%)		
Stenosis (60–100%) and stented	1 (0.5%)		
**Posterior descending artery (PDA)**			
Patent (no stenosis)	213 (96.8)		
Stenosis (30–70%) without stented	6 (2.7%)		
Stenosis (60–100%) and stented	1 (0.5%)		

Significant positive moderate correlations were found between HC and the number of stented coronary arteries and number of severe stenosed coronary arteries, with correlation coefficients of 0.5 and 0.4, respectively. Further, a significant positive moderate correlation was found between WC and the number of stented coronary arteries (correlation coefficient 0.4). Meanwhile, weight, WC, WHtR, and BAI were found to have significant positive weak correlations with the number of severe stenosed arteries. WHtR and BAI were also found to have significant positive weak correlations with the number of stented arteries. A non-significant negative correlation was identified between WHR and the number of severe stenosed arteries and number of stented arteries (see Table 3).

**Table 3:** Correlations (Pearson) between obesity parameters and number of stenosed coronary arteries (≥ 60%) and number of stented coronary arteries (*N* = 220).

	Number of severe stenosed arteries (≥ 60%)	P value	Number of stented arteries	P value
Weight kg	0.16	**0.02**	0.16	0.18
Waist circumference	0.29	** > 0.001**	**0.4**	** > 0.001**
Hip circumference	**0.4**	** > 0.001**	**0.5**	** > 0.001**
Waist height ratio	0.22	**0.001**	0.31	** > 0.001**
Waist hip ratio	− 0.12	0.11	− 0.17	0.01
Body adiposity index	0.25	** > 0.001**	0.32	** > 0.001**
Body mass index	0.03	0.67	0.06	0.37
Body shape index	− 0.03	0.63	− 0.01	0.84

Significant values are in bold.

The ability of the models to explain the variance in the number of stented coronary arteries was assessed, as shown in Table 4. Model (1), which had one predictor (HC), explained 25.9% of the variance in the number of severe stented coronary arteries (P < 0.001), whilst Model (5) (HC, HS-CRP, triglycerides, smoking) explained 34.4% (P = 0.02). Model 5 illustrated that an increase in HC by 6.2 cm (β (0.465) × SD (13.4)), HS-CRP by 0.26 mg/dl (β (0.183) × SD (1.43)), serum triglycerides level by 6.3 mg/dl (β (0.184) × SD (34.3)), or daily cigarette consumption by 1.6 (β (0.138) × SD (11.5)) would lead to an increase in the number of stented coronary arteries by one (see Table 4).

**Table 4:** Forward linear regression between obesity parameters and socio-demographic and health variables, and the number of stented coronary arteries (*N* = 220).

Model		R^2^ for the model (P)	Standardized coefficients (β)	P	CI 95% LL	UL
1	HC (m)	0.259 (< 0.001)	0.509	** < 0.001**	0.435	0.707
2	HC (m)	0.296 (0.002)	0.496	** < 0.001**	0.423	0.69
	HS-CRP (mg/dl)		0.193	**0.002**	0.171	0.714
3	HC (m)	0.325 (0.004)	0.471	** < 0.001**	0.396	0.66
	HS-CRP (mg/dl)		0.172	**0.004**	0.125	0.663
	Triglycerides (mg/dl)		0.174	**0.004**	0.032	0.172
4	HC (m)	0.344 (0.02)	0.465	** < 0.001**	0.391	0.652
	HS-CRP (mg/dl)		0.183	**0.002**	0.152	0.685
	Triglycerides (mg/dl)		0.184	**0.002**	0.039	0.178
	Smoking (cigarettes/day)		0.138	**0.02**	0.013	0.153

The last model explained 34.4% of the variation of frailty score (P = 0.02).

Significant values are in bold.

The ability of the models to explain the variance in the number of stenosed coronary arteries was also assessed, as shown in Table 5. Model (1), which had one predictor (HC), explained 14.7% of the variance (P < 0.001), whilst Model (5), which had five predictors (HC, smoking, HS-CRP, HDL, and triglycerides), explained 25.7%. Model 5 illustrated that an increase in HC by 4.4 cm (β (0.331) × SD (13.4)), daily cigarette consumption by 2.4 cigarettes (β (0.208) × SD (11.5)), or HS-CRP by 0.24 mg/dl (β (0.165) × SD (1.43)) would lead to an increase in the severity of stenosis of the coronary arteries by 1% (see Table 5).

**Table 5:** Forward linear regression between obesity parameters and socio-demographic and health variables, and the number of severe stenosed coronary arteries (≥ 60%) (*N* = 220).

Model		R2 for the model (P)	Standardized coefficients (β)	P	CI 95% LL	UL
1	HC (m)	0.147 (< 0.001)	0.383	** < 0.001**	0.294	0.597
2	HC (m)	0.18 (0.005)	0.379	** < 0.001**	0.292	0.589
	Smoking (cigarettes/day)		0.182	**0.005**	0.034	0.191
3	HC (m)	0.218 (0.002)	0.366	** < 0.001**	0.28	0.571
	Smoking (cigarettes/day)		0.199	**0.002**	0.045	0.201
	HS-CRP (mg/dl)		0.196	**0.002**	0.165	0.762
4	HC (m)	0.239 (0.021)	0.35	** < 0.001**	0.262	0.552
	Smoking (cigarettes/day)		0.198	**0.002**	0.045	0.199
	HS-CRP (mg/dl)		0.18	**0.005**	0.13	0.723
	HDL (mg/dl)		− 0.147	**0.021**	− 0.587	− 0.048
5	HC (m)	0.257 (0.031)	0.331	** < 0.001**	0.239	0.53
	Smoking (cigarettes/day)		0.208	**0.001**	0.052	0.205
	HS-CRP (mg/dl)		0.165	**0.01**	0.96	0.687
	HDL (mg/dl)		− 0.141	0.26	− 0.572	− 0.036
	Triglycerides (mg/dl)		0.135	0.36	0.006	0.159

The last model explained 25.7% of the variation of frailty score (P = 0.031).

Significant values are in bold.

## Discussion

This study is the first to comprehensively compare between the seven traditional obesity parameters in terms of their role in predicting CAD among patients undergoing cardiac catheterization. Our study results revealed a moderate correlation between HC and the number of stented coronary arteries, with a cut-off value of 103 cm. Furthermore, HC had significant regression levels with the number of stented coronary arteries and the number of severe stenosed coronary arteries. The second-best obesity parameter for predicting CAD was WC, as shown by the moderate correlation between WC and the number of stented coronary arteries (cut-off value of 95 cm). This finding supports the findings of previous studies which reported WC as being the best indicator of the risk factors for CAD (i.e., hyperlipidemia, hypertension, hyperglycemia)^9–11^.

On the other hand, Mornar Jelavić et al.^12^ found that WHR, followed by WHtR, was more superior than BMI in significantly predicting the stenosis of coronary arteries (≥ 60%). Similarly, Jelavic et al.^13^ found that WHR (≥ 0.90 m) had a significantly correlation with the severity of stenosed coronary arteries (stenosis percentage ≥ 70%). This was attributed to the fact that WHR measures central obesity, which has been revealed to be negatively associated with the health status of patients with CAD. On the other hand, Gregory et al.^14^ found no statistically significant difference between the BMI categories in terms of their association with the severity of CAD (i.e., an increase in the number of diseased coronary arteries and their stenosis percentage) or CAD-related mortality. Gregory et al.^14^ explained their results by highlighting that there may be other risk factors which place patients at a higher risk of developing CAD. They also explained that physicians may suspect CAD among obese patients earlier than among non-obese patients and may therefore pay closer attention to obese patients with regards to the development of CAD or its related symptoms.

However, there was no agreement in the literature^12–14^ with regards to the best obesity parameter for predicting CAD (either stenosis or percentage) or its related symptoms. It has been evidenced in the literature that central obesity is a leading cause of the development of CAD or its related symptoms^20–22^. Central obesity is characterized by the increase in abdominal girth, which is evidenced by the increase in WC, HC, WHR, and WHtR^20^. Wan et al.^20^ found that central obesity was associated with increased tendency to have CAD. Their results indicated that the increase in central obesity was associated with the increase in level of LDL and triglycerides, which is strongly associated with CAD. In accordance with these results, our study revealed that the central obesity measure HC was the best possible predictor of CAD, followed by WC. Central obesity may explain the findings reported in the literature^12,13^, which indicated relationships between “WHR and WHtR” and CAD.

With regards to the role of heath variables in predicting CAD, our findings indicated that HS-CRP had a significant positive regression level with CAD (i.e., the number of stented coronary arteries and number of severe stenosed coronary arteries (≥ 60%)). These findings are in concordance with five previous studies^23–27^ which reported a positive association between the increase of serum level of high sensitive CRP and the incidence of CAD. This finding was explained by the fact that CRP is secreted by hepatocytes, where its synthesis is regulated by cytokines. It is known that cytokines regulate the growth of body immune cell in response to inflammation. Further, HS-CRP assay measures low levels of CRP, and the elevation of HS-CRP in CAD patients may result from inflammation within the atherosclerotic plaque.

In the present study, smoking was found to be significantly associated with higher numbers of stented coronary arteries and severe stenosed coronary arteries (≥ 60%), which comes consistent with the studies of Song et al.^28^, Bouabdallaoui et al.^29^, and Chen et al.^30^. These studies attributed this finding to the fact that smoking may increase oxidative stress, which is considered a precursor for atherosclerosis. They emphasized that smoking contributes to CAD not only through the production of reactive oxygen radicals in smoke, but also through weakening the antioxidant defense systems.

Meanwhile, Nardin et al.^31^ held that smoking was not a contributing factor of CAD, and Akyüz et al.^32^ found that nonsmokers had a higher tendency than smokers to develop severe CAD. Akyüz et al.^32^ explained this finding by highlighting that non-smoker CAD patients have higher tumor necrosis factor (cytokine 1A (TL1A)). TL1A is a member of the tumor necrosis factor (TNF) superfamily. It has been found to be associated with the death domain receptor 3 (DR3) and to increase with inflammation (novel indicator of atherosclerosis). TL1A is considered a promising biomarker for diagnosing CAD and indicating CAD lesion complexity.

The current study found that the increase in serum triglyceride level had a significant association with the number of stented coronary arteries. Correspondingly, three previous studies^33–35^ found a positive relationship between the increase in serum level of triglycerides and CAD. Talayero and Sacks^36^ explained that several species of triglyceride-rich lipoproteins (TRLs), including very low-density lipoprotein (VLDL), VLDL remnants, and chylomicron (CM) remnants, appear to promote atherogenesis independently of LDL. These remnants are subject to endothelial accumulation and uptake by macrophages to form foam cells. In turn, these foam cells promote fatty streak formation, which is a precursor of atherosclerotic plaque.

## Conclusion

Among the seven traditional obesity parameters, hip circumference was found to have the most significant association with the number of stenosed coronary arteries (≥ 60%) among patients with CAD undergoing cardiac catheterization. HS-CRP, triglycerides, and smoking had significant positive associations with CAD (the number of stenosed coronary arteries (≥ 60%) and number of stented coronary arteries). Healthcare providers, including nurses, need to be made aware of a set of characteristics (i.e., increased serum level of triglycerides, HS-CRP, and smoking) which may predict the incidence of CAD, particularly the number of stenosed coronary arteries (≥ 60%) and number of stented coronary arteries, among patients with CAD undergoing cardiac catheterization.

## Data Availability

Data can be requested from the first author upon reasonable request.
